# Microbeam radiotherapy – clinical and dosimetric investigation of a first clinical application

**DOI:** 10.1186/s13014-026-02946-2

**Published:** 2026-10-23

**Authors:** Kim Melanie Kraus, Liam R. Day, Jan J. Wilkens, Stephanie E. Combs, Stefan Bartzsch

**Affiliations:** 1 Department of Radiation Oncology, TUM School of Medicine and Health, TUM University Hospital, Technical University of Munich, 81675 Munich, Germany; 2 Institute for Advanced Study, Technical University of Munich (TUM), Garching, Germany; 3 Partner Site Munich, German Consortium for Translational Cancer Research (DKTK), 80336 Munich, Germany; 4 CollimateHealth GmbH, Haager Str. 5, Munich, Germany

**Keywords:** Microbeam radiation therapy, Spatially fractionated radiotherapy, Treatment planning, Dose calculation

## Abstract

**Background:**

Microbeam Radiotherapy (MRT) is an emerging radiotherapy technique which delivers spatially fractionated beams on a micrometre scale. In preclinical studies MRT has demonstrated superior normal tissue sparing compared to conventional radiotherapy while maintaining tumour control. However, MRT has traditionally been achievable only at large synchrotron facilities which has impeded clinical translation. The development of a compact line-focus X-ray tube (LFXT) provides a pathway for clinical MRT, but identification of clinically and dosimetrically suitable patient scenarios is required for first-in-human trials.

**Materials and methods:**

Potentially eligible patients were selected from our patient cohort based on clinical criteria including palliative treatment indication, superficial tumour location, reproducible and safe positioning, minimal interference from other therapies, and expected benefit from MRT. Three-dimensional forward treatment planning was performed in Eclipse using gantry rotations, collimator positioning, and conformal masks to treat the clinical target volume (CTV). Dose was calculated in Eclipse using a custom Monte Carlo–based hybrid algorithm adapted for the compact LFXT system with a 300 kVp beam and an eccentric focal spot. A single-fraction equivalent uniform doses (EUD) of 3 Gy was prescribed to the target volume. Dose distributions were analysed using dose-volume histograms (DVHs), peak and valley depth dose profiles, and peak-to-valley dose ratios (PVDRs).

**Results:**

Selected cases included primary lymphoma, squamous cell carcinoma, and superficial soft tissue metastases. Dosimetric analysis under exploratory assumptions demonstrated dosimetrically feasible treatment plans with optimal target coverage and organ-at-risk (OAR) sparing for gluteal, nuchal, and inguinal tumours. Maximum OAR doses remained below 8.5 Gy, except in bone or overlapping high-density regions. The mean peak and valley dose across all plans was 57.2 Gy and 3.9 Gy, respectively, with a mean PVDR of 15.4. Dose inhomogeneities were observed due to tissue heterogeneity, kilovoltage beam interactions, and a limited range of suitable gantry angles. Tumours in anatomically complex regions, including those in proximity to OARs or tissue inhomogeneities, showed less favourable trade-offs between target coverage and OAR sparing.

**Conclusion:**

This study identifies clinically and dosimetrically suitable scenarios for first-in-human MRT trials, highlighting superficial tumours as optimal initial indications. While peak, valley, and PVDR values align with preclinical evidence, their clinical significance remains uncertain. Careful, safety-driven dose escalation studies will be required to establish a foundation for responsible and effective clinical application of MRT in early-phase clinical trials.

## Introduction

Microbeam Radiotherapy (MRT) is an innovative technique employing spatially fractionated beams on the micrometre scale to deliver highly heterogeneous dose distributions to the tumour. MRT is steadily advancing towards clinical translation. Preclinical studies have consistently demonstrated remarkable normal tissue sparing without compromising tumour control [1–5]. A broad spectrum of biological effects have been reported including alterations in tumour vasculature [6–8], modulation of immune responses [9, 10], and changes in cellular signalling pathways [11].

Despite these promising findings, significant technical challenges have so far limited clinical implementation. Traditionally, large synchrotron facilities have been required to generate microbeams with sufficiently steep penumbrae and ultra-high dose rates (required to maintain spatial fractionation), thereby restricting widespread clinical use. In contrast, minibeam radiation therapy, which imposes less stringent physical requirements, has already demonstrated successful clinical application [12].

To overcome these technical barriers and enable clinical translation of MRT, a pre-clinical prototype of a compact line-focus X-ray tube (LFXT) has been developed [13, 14]. The feasibility of MRT dose calculation and treatment planning using this system has been successfully demonstrated [15–17]. For initial application within a clinical trial however, appropriate treatment scenarios must be carefully defined. This requires careful consideration of both technical parameters (such as beam energy, patient positioning, and delivery technique) and clinical factors (including treatment indication, tumour location, and dose prescription).

Treatment planning studies were performed to assess the technical feasibility and dosimetric applicability of MRT using the LFXT in order to identify suitable clinical scenarios, thereby establishing a rational pathway toward first-in-human MRT studies.

## Materials and methods

### Patient data

Potentially suitable cases were selected from the patient cohort at TUM University Hospital’s Department of Radiation Oncology. Selection criteria included:

- Palliative radiotherapy treatment indication and/or potential benefit from MRT.
- Measurable and limited side effects from radiation.
- No potentially interfering therapy.
- Reproducible and safe patient positioning, including consideration of the vicinity of organs at risk (OARs).
- Superficial target location.

Radiation treatment data included planning Computed Tomography (CT) image sets with delineated target and OAR volumes.

### Dose calculation and treatment planning

#### Dose calculation algorithm

MRT peak doses are comprised mainly of energy deposited by short-ranged electrons generated in primary photon interactions, while the valley dose is comprised mainly of the energy absorbed from scattered photons. MRT dose distributions were calculated using a Monte Carlo (MC) based, dose-to-medium algorithm called the Hybrid [15]. The algorithm combines an accurate MC simulation of photon interactions in the orthovoltage energy range with an efficient electron scatter kernel convolution to calculate the microbeam dose distribution. For each CT voxel, the Hybrid algorithm calculates the 1D microbeam dose profile at resolutions of 1 μm and reports the peak dose and the valley dose per field. The algorithm was implemented in a research version of the commercially available treatment planning software Eclipse v16.1 (Varian Medical Systems, Palo Alto, CA). MC simulations were performed using 2 × 10^8^ particles per field. Photons were initialised at the field position immediately before the tumour conforming block. The size of the field was defined by the rectangle fitted to the tumour conforming block’s aperture.

MC uncertainties were calculated per voxel using the history-by-history method [18]. An average plan uncertainty per field was calculated as the average uncertainty in voxels having dose above half the maximum dose. Note that an additional CT number threshold between − 200 HU and 200 HU was applied when calculating the plan uncertainty since the dose-to-medium calculation for low energy photons causes large dose spikes in air and high-density bone which would otherwise obscure the reporting of the uncertainty in water-like tissues.

The 1σ MC plan uncertainties ranged between approximately 3–4% for the primary dose (or peak dose) and 5–6% for the secondary dose (or valley dose), per field. The dose calculation grid resolution matched the CT resolution and ranged from approximately 1 × 1 × 2 mm^3^ to 1 × 1 × 3 mm^3^.

The microbeam geometry in the Hybrid algorithm was modelled by an ideal, perfectly attenuating multi-slit collimator (MSC) having 50 μm wide microbeams separated by 400 μm centre-to-centre distances at the MSC position. The tumour conformal block was also modelled as a perfectly attenuating binary mask.

The design of the MSC for the LFXT is planned to be 14 mm thick DENSIMET^®^ and transmission through the MSC is therefore expected to be negligible. The tumour conforming block will be made of 15 mm thick Cerrobend and transmission through the block is similarly expected to be negligible. Photon scattering off of the MSC and tumour conforming block is not currently accounted for in the dose calculation and consequently calculated doses will likely be underestimated, especially in the valley dose at the surface where phantom scatter is less prominent.

Beam divergence for the MC part of the Hybrid algorithm was handled by independently sampling both the LFXT source distribution, and the photon’s initial position at the field plane, and calculating the direction vector between them. Divergence during the electron dose calculation stage of the Hybrid algorithm was handled simply by broadening the 1D microbeam dose profile by a magnification factor equal to the source-to-voxel distance divided by the source-to-MSC distance. An additional Gaussian profile is convolved with the 1D microbeam dose profile to account for blurring of the microbeam peak doses due to the LFXT focal spot size (details of the Gaussian source correction are presented by M. Ahmed, et al. [19]).

### Source implementation

**Fig. 1:**
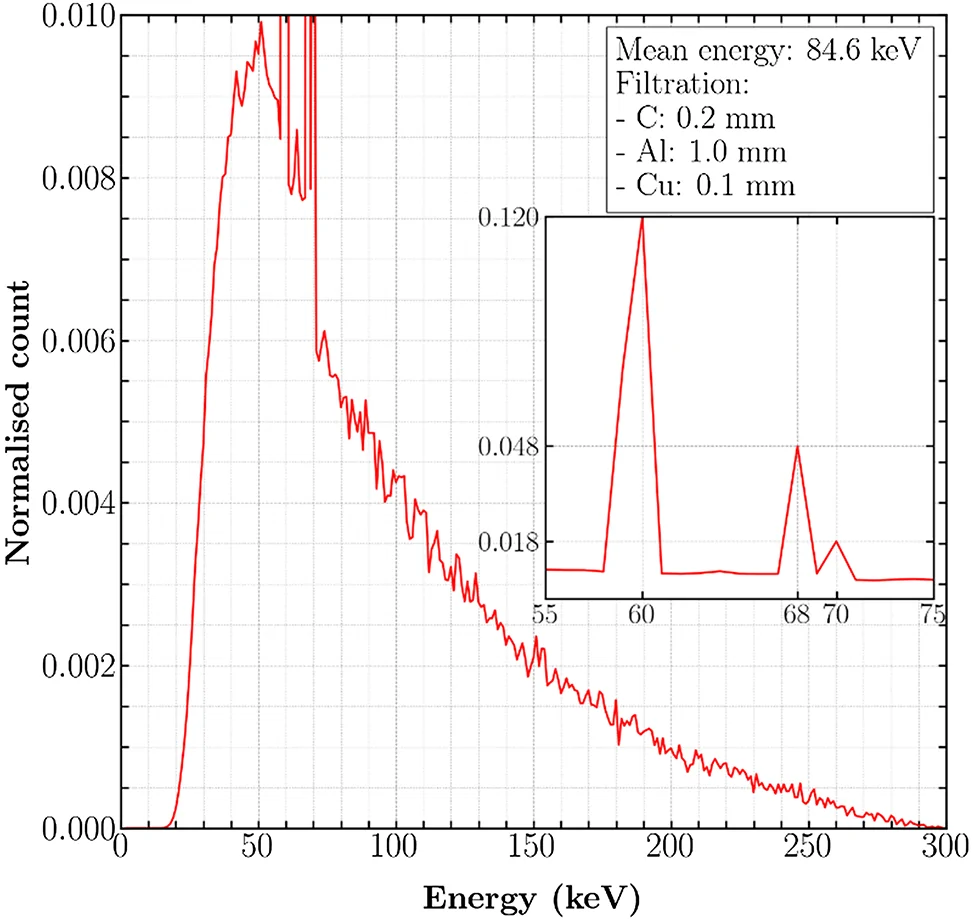
Energy spectrum of the LFXT [14]

The Hybrid dose calculation algorithm was adapted for use with the compact LFXT system and implemented into the Eclipse treatment planning platform. The LFXT, as a compact X-ray source, employs an eccentric beam focal spot. The beam energy matched the anticipated spectral range for a 300 kVp electron tube potential, with additional filtration including 0.2 mm C (to account for the diamond window), 0.1 mm Cu, and 1.0 mm Al (see Fig. 1).

### 3D treatment planning

The LFXT was defined in Eclipse as a custom linac treatment machine using the standard treatment geometry of a linac including the gantry, collimator, and table. The range of machine parameters (including field size, gantry angle, collimator angle – which defines the orientation of the microbeams, and couch translation/rotation) were left unrestricted in order to facilitate ideal planning for clinical treatment, exploration of dosimetric potential, and to identify necessary design changes in the construction of the LFXT. However, it should be noted that the first clinical LFXT prototype will have additional restrictions on beam orientation and positioning compared to conventional linacs. Machine parameters specific to the LFXT including the source-to-MSC distance (set to 40 cm), microbeam width and spacing (50 μm and 400 μm, respectively), and spectral filtration (as in Fig. 1), were defined within the Hybrid algorithm options in Eclipse.

3D forward treatment planning was performed using two fixed, coplanar beam positions with gantry angles manually selected to achieve good target coverage while reducing dose to healthy tissue. No automatic fluence or dose optimisation was applied. The isocenter was defined to match the centre of mass (COM) of the CTV and was repositioned inside the tumour volume if required. The microbeam collimator was rotated by 90° between the two fields to achieve a cross-firing beam geometry. Field widths were up to 20 cm wide. A conformal mask of the target volume was positioned close to the patient surface while avoiding overlap.

### Dose prescription and analysis

The optimal dose prescription for MRT is currently unknown due to the absence of clinical application. To date, it is not clear which volume to plan for, nor is it clear whether the peak, valley, or homogeneous dose should be prescribed. The equivalent uniform dose (EUD) may be suitable for comparing MRT and uniform dose distributions [20]. For dose analysis therefore, the heterogeneous physical dose was converted to the EUD per voxel [21], assuming that it produces the same biological effect as a uniformly delivered dose. A 3 Gy EUD was prescribed to cover 95% of the target volume, simulating a single fraction as a potential replacement for a conventional radiotherapy treatment regimen. Conventional planning target volumes (PTVs) often include margins of several millimetres that may not reflect the required accuracy for MRT dose delivery. Therefore, the clinical target volume (CTV) defined for conventional radiotherapy was used as the target volume to prevent high peak doses in healthy tissue (assuming 100% positional accuracy). The EUD, and the peak and valley doses corresponding to the prescribed EUD, were analysed. Whereas peak doses are assumed to cause local tissue destruction within the tumour and result in local tumour control, intermediate valley doses contribute to normal tissue toxicity. Doses to OARs were evaluated using dose-volume histograms (DVHs).

Dose constraints for OARs for MRT are also unknown. Therefore, the known dose constraints for single- or hypofractionated dose delivery in conventional radiotherapy were used to estimate the OAR constraints. For target coverage, the minimum EUD to 2%, 95%, and 98% of the volume, as well as the mean EUD, were analysed. Additionally, the peak dose, valley dose, and peak-to-valley dose ratio (PVDR) were reported with depth.

## Results

A retrospective screening of our internal patient database identified 12 representative cases for MRT dosimetric evaluation. The cohort included both favourable and anatomically challenging scenarios. Target coverage was high (EUD₉₅_%_: 95.4–105.8% of prescription dose), with mean cumulative peak and valley doses of 57.2 ± 30.7 Gy (25.7–151.0 Gy) and 3.9 ± 1.5 Gy (2.4–7.6 Gy), respectively, yielding a mean PVDR of 15.4 ± 6.4 (6.8–29.4). Maximum OAR EUDs were < 8.5 Gy for nearly all organs, demonstrating the feasibility of achieving target coverage while limiting OAR exposure.

### Patient data

Our internal database was screened for patients with treatment indications for superficial tumours (including lymphoma, sarcoma, metastases, skin tumours, and tumours in the head and neck) with a palliative treatment intention or an expected potential benefit from MRT. The data were intentionally enriched for favourable MRT and Phase 1 scenarios, including superficial tumour locations with no major OARs in proximity and the feasibility of fixation for therapy. These tumour sizes can be covered by the expected field sizes without need for concomitant systemic antitumoural therapy, such as immunotherapy or chemotherapy.

A range of histologies and tumour locations were included to avoid limiting the study to a single predefined histology, and to assess dosimetric feasibility for tumours in proximity to critical structures. From a preselected cohort of 35 patients, we selected 12 patients covering a range of diseases including primary lymphoma, squamous cell carcinoma, a soft tissue metastasis of an SCLC, lymph node metastases of a malignant melanoma, and a penis carcinoma (see Table 1).

Lymphoma patients were chosen because lymphoma is known to be radiosensitive, requiring only low radiation doses, making it a potential candidate for MRT. Superficial occurrences at reliably positionable locations were chosen. These tumour sites include locations in the head which can be safely fixated such as preauricular, orbital, nuchal or at the capillitium; as well as tumours in the gluteal and inguinal region which are relatively distant from major OARs. All of the above tumours were located superficially, with skin toxicity among the most likely toxicities to be detected and quantified early. The orbital lymphoma case was selected as a dosimetrically and clinically challenging case with radiosensitive structures close to the target volume and with limited therapeutic alternatives.

Two squamous cell carcinoma cases were also included. One tumour occurred in the inner corner of the eye, which was already blind, and was close to sensitive structures. This might represent a dosimetrically challenging patient case with limited alternative treatment options and potential benefit from MRT. The other squamous cell carcinoma was located in the preauricular region; a superficial tumour location in the head and neck region which is easy to position, with toxicity of the skin being an expected early and measurable side effect.

One gluteal, superficial soft tissue metastasis of a Small Cell Lung Cancer (SCLC) patient with multiple metastases and one inguinal lymph node metastasis of a penis carcinoma were also chosen. These tumour sites are separated from critical OARs which is favourable for MRT using a small number of fields. These patient cases represent a common palliative radiation treatment indication, but in the selected cases, no major early side effects are observed. Skin toxicity is expected and potential side effects to lymphatic drainage are noted.

### Dose data

**Table 1:** Volume and dose data for Clinical target volumes (CTVs)

Case	Treatment indication	Structure	V[cm^3^]	EUD_min_ [Gy]	EUD_98%_ [Gy]	EUD_95%_ [Gy]	EUD_2%_ [Gy]	EUD_mean_ [Gy]	EUD_max_ [Gy]
1	Soft tissue metastasis of an SCLC in the gluteal region	CTV	76.1	1.9	2.8	3.0	4.7	3.6	6.2
		Os ilium						0.7	6.1
		Myelon						0.1	2.1
		Femoral head ipsilateral						0.5	2.1
2	Diffuse DLBCL gluteal	CTV	250.8	0.0	2.8	3.0	4.6	3.8	6.6
		Femoral head ipsilateral						0.6	5.8
		Femur ipsilateral						0.4	4.1
		Sigma						0.4	2.0
3	LN metastasis of MM nuchal	CTV	44.6	1.7	2.8	3.0	4.1	3.5	12.9
		Parotid Gland ipsilateral						0.5	2.3
		Bulbus ipsilateral						1.1	1.7
4	CLBCL pre-auricular	CTV	57.3	0.0	2.8	3.0	5.4	3.7	12.0
		Cochlea ispilateral						1.4	2.3
		Lacrimal Gland ipsilateral						1.4	1.8
		Parotis ipsilateral						3.3	6.0
		Brain						0.4	8.5
5	Cutaneous NHL capillitium	CTV	21.9	0.0	2.4	3.1	12.4	5.4	19.1
		Skull						1.2	21.3
		Optic nerve ipsilateral						0.9	3.4
6	SCC of the inner corner of the eye	CTV	57.8	0.0	0.0	3.2	12.5	5.0	17.0
		Lens ipsilateral						4.9	5.6
		Lacrimal Gland ipsilateral						4.7	5.6
		Brain						0.1	4.1
		Bulbus ipsilateral						4.0	5.3
7	MALT lymphoma of the orbita	CTV	55.2	0.0	2.8	3.0	9.4	4.2	11.4
		Chiasm						1.2	2.2
		Lens ipsilateral						4.0	5.3
		Retina ispislateral						4.5	5.2
		Optic nerve ipsilateral						3.7	5.0
		Optic nerve contralateral						1.3	2.3
		Brain						0.1	4.8
8	Cutaneous TCL of the ear helix	CTV	49.3	0.0	2.6	2.9	5.4	3.8	12.7
		Mandible						0.6	6.8
		Parotid Gland ipsilateral						2.8	6.1
		Cochlea ispilateral						1.4	2.1
		Bulbus ipsilateral						0.3	1.1
		Brain						5.0	17.0
9	SCC of the ear	CTV	28.4	1.5	2.6	2.9	9.3	4.6	12.1
		Eye ipsilateral						0.3	1.2
		Mandible						0.8	12.1
		Myelon						0.1	1.0
		Parotid Gland ipsilateral						2.2	9.4
10	Follicular Lymphoma inguinal	CTV	145.3	0.0	2.7	3.0	6.5	4.8	21.8
		Bladder						0.9	6.6
		Bowel						0.0	0.7
		Femoral head ipsilateral						2.5	6.8
		Rectum						0.3	1.6
11	Lymph node metastasis of a penis carcinoma inguinal	CTV	553.5	1.5	2.7	2.9	5.5	4.2	21.0
		Bladder						1.3	4.4
		Bowel						0.1	1.1
		Femoral head ipsilateral						1.0	3.8
		Rectum						0.3	1.0
		Penile Bulb						0.8	3.1
12	SCC pre-auricular	CTV	22.2	0.0	2.5	2.9	9.8	5.5	19.0
		Brain						0.4	9.7
		Mandible						0.4	8.2
		Cochlea ispilateral						2.7	4.4
		Lacrimal Gland ipsilateral						2.2	4.2
		Nervus opticus ipsilateral						0.7	1.7
		Lens ipsilateral						0.7	1.5
		Eye ipsilateral						0.8	2.7

CTV volumes (V), minimum (min), mean (mean), and maximum (max) equivalent uniform doses (EUD) are reported, along with the doses to 98%, 95%, and 2% of the CTV

Abbrevaitions: DLBCL - diffuse large B-cell lymphoma, LN - lymph node, MM - malignant melanoma, CLBCL - cutaneous large B-cell lymphoma, NHL - Non-hodgkin lymphoma, SCC - squamous cell carcinoma, MALT - mucosa-associated lymphoid tissue tumour, TCL - for T-cell lymphoma

**Table 2:** Centre of mass peak, valley doses and PVDRs for all patient cases

Case	Peak dose [Gy]	Valley dose [Gy]	PVDR
1	42.8	3.6	12.1
2	28.0	2.7	10.5
3	60.7	2.5	24.5
4	47.3	2.4	19.5
5	63.0	7.6	8.3
6	64.4	4.3	14.8
7	42.8	3.0	14.5
8	49.2	3.3	14.7
9	54.0	2.8	19.2
10	57.6	5.3	10.9
11	25.7	3.8	6.8
12	151.0	5.1	29.4
**Min**	25.7	2.4	6.8
**Max**	151.0	7.6	29.4
**Mean**	57.2	3.9	15.4

**Fig. 2:**
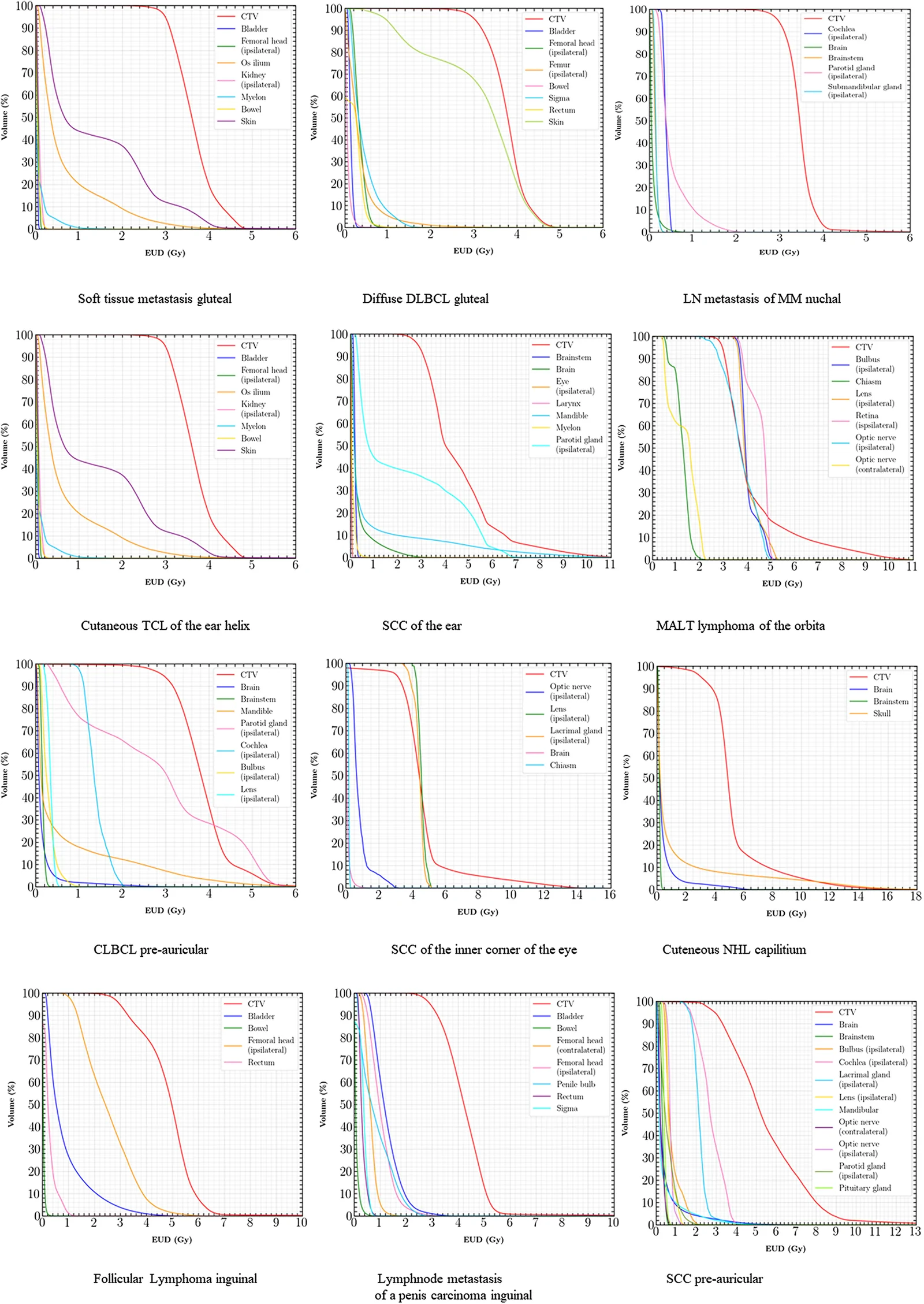
DVHs showing EUD for all investigated cases. The top row represents the dosimetrically best cases

**Fig. 3:**
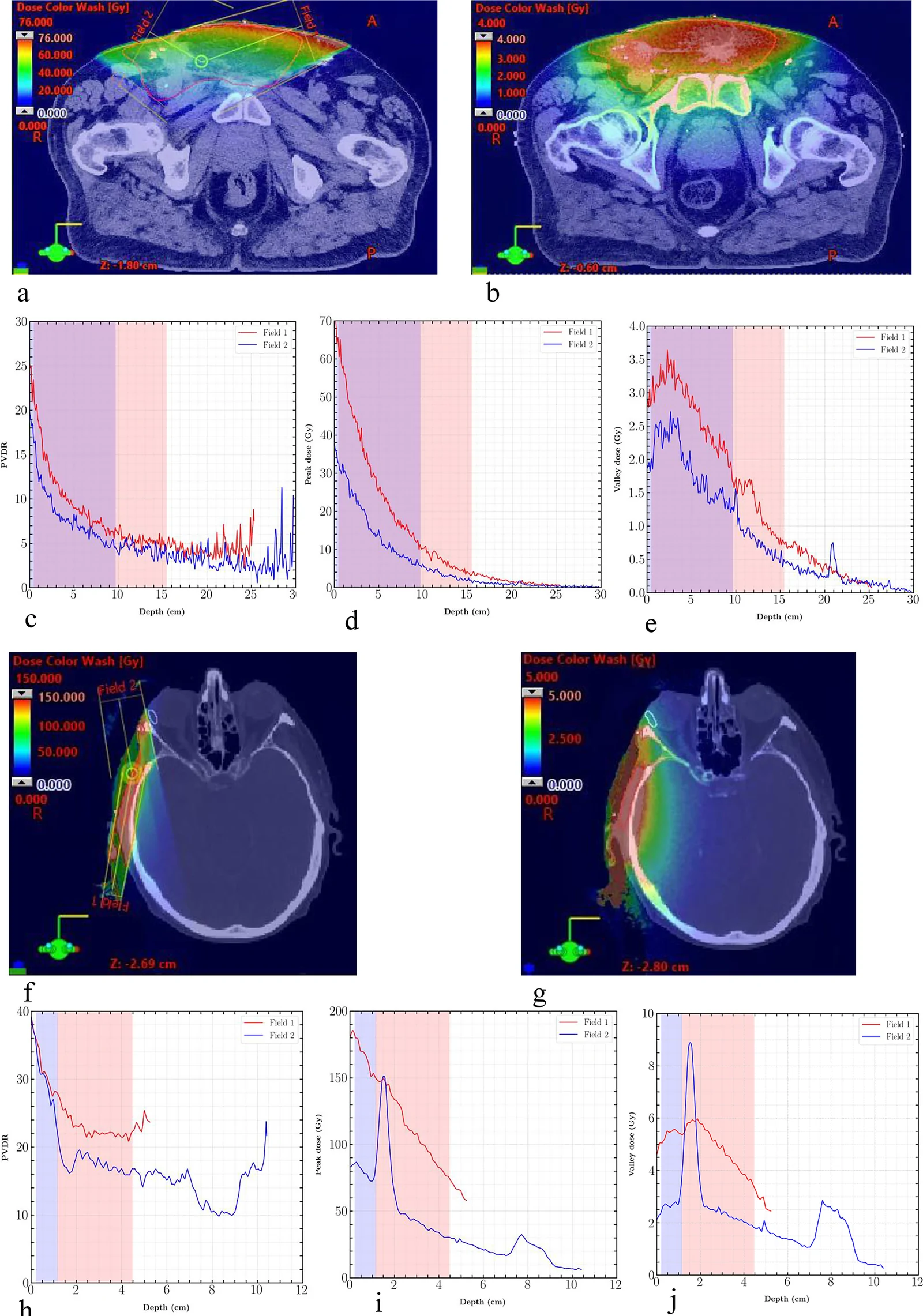
Peak and valley dose distributions for cases 11 (**a**, **b**) and 12 (**f**, **g**). The corresponding PVDR (**c**, **h**), peak (**d**, **i**) and valley dose curves (**e**, **j**) are shown as functions of depth through the centre of mass (COM) of the target volume. The surpassed tumour volume is highlighted in red for field 1 and in blue for field 2. PVDRs decrease with depth depending on the density of the tissue traversed, and peak doses are highest near the surface. The very high peak dose in graph f results from a high-atomic-number bony structure within the target volume

The most favourable trade-off between target coverage and organ-at-risk (OAR) sparing was observed for two gluteal and a nuchal tumour (case numbers 1, 2 and 3 in Table 1, see also Fig. 2 top row). Also, the two inguinal tumour cases (case numbers 10 and 11) showed a good compromise between the target EUD and the EUD to OARs. Generally, dose distributions were highly inhomogeneous. Minimum target EUDs ranged from 0 Gy (reflecting regions containing air) to 1.9 Gy, while maximum target EUDs exceeded 6 Gy in all cases. However, EUD₂_%_ remained below 6 Gy for most plans. Target coverage (EUD₉₅_%_) ranged from 95.4% to 105.8% of the prescribed dose. Small deviations from 3 Gy for the EUD_95%_ were due to differences in the DVH calculation between the Eclipse treatment-planning software and the internal dose calculation algorithm. For consistency, we decided to report only Eclipse-derived values. Maximum OAR EUDs were below 8.5 Gy for nearly all organs except for bony structures, and structures that overlapped bone, due to the enhanced interactions of kV radiation in high-density tissue. The mean cumulative peak and valley doses were 57.2 ± 30.7 Gy (range 25.7–151.0 Gy) and 3.9 ± 1.5 (range 2.4–7.6 Gy), respectively, with a mean peak-to-valley dose ratio (PVDR) of 15.4 ± 6.9 (range 6.8–29.4) (see Table 2). Peak, valley, and PVDR depth dose profiles through the COM of the target volume for case 11 (having a low cumulative COM peak dose of 25.7 Gy) and for case 12 (having a high cumulative COM peak dose of 151.0 Gy) are shown in Fig. 3.

## Discussion

This study uses real patient data to identify clinically reasonable and dosimetrically suitable target indications for the first clinical application of MRT within a phase I trial.

Using a predefined set of clinically relevant selection criteria, a cohort of potentially eligible patient cases was identified. Key selection criteria included treatment intent, tumour location, and the feasibility of patient immobilisation. Tumours of various histologies were analysed since the current state of evidence does not indicate increased efficacy by histology. However, some of the histologies investigated in this study have been successfully studied at the preclinical level [22–24]. Additionally, patients were selected with regard to measurable and limited side effects with a focus on skin toxicity. Patients receiving concomitant antitumoural systemic therapy such as chemotherapy or immunotherapy were excluded. Clearly, this retrospective study focuses on the dosimetric feasibility using a compact MRT device and can only estimate expected side effects due to simulated dose values.

When analysing and interpreting MRT dose distributions and DVHs, essential differences from conventional megavoltage radiotherapy must be considered. While dose heterogeneity is usually minimised for megavoltage radiotherapy, it is the key concept underlying the MRT effect. Two distributions with substantially different microscopic organisation can potentially produce similar DVH summaries. Thus, conventionally used dose parameters and DVHs should be used with caution since a conventional DVH collapses spatial information into a volume-dose relationship. Commonly used dosimetric parameters such as the mean, minimum, and maximum doses must be interpreted in the context of spatial heterogeneity for MRT, and additional parameters such as peak and valley dose are crucial for dose reporting and interpretation.

The dosimetric analysis performed in this study demonstrated that, under exploratory assumptions, dosimetrically feasible treatment plans can be achieved within standard dose constraints commonly applied in conventional radiotherapy. The most favourable dosimetric results were obtained for gluteal tumour locations. Tumours located in the nuchal and inguinal regions also exhibited a reasonable trade-off between target coverage and sparing of OARs. These findings are primarily attributable to the superficial tumour location and the spatial separation from critical healthy structures. Thus, the expected toxicity is low. Altogether, the dosimetric and clinical characteristics of these patient cases provide a reasonable scenario for the first clinical application of MRT. In contrast, dose distributions for tumours in more anatomically complex regions, such as the orbital or pre-auricular areas, were dosimetrically inferior due to proximity to, or direct overlap with, critical OARs.

When compared with conventional radiotherapy DVHs, the simulated MRT plans (which report the dose-to-medium) exhibited pronounced dose enhancement. The dose enhancement arises from the increased photoelectric absorption cross-section of low energy photons in high density tissues. Consequently, voxels of bone can exhibit exceptionally high local doses compared to conventional dose plans which typically report dose-to-water.

The mean COM peak dose, valley dose, and PVDR were approximately 57 Gy, 4 Gy, and 15, respectively, with absolute maximum peak doses reaching up to approximately 151 Gy and maximum valley doses of up to 7.6 Gy. To date, no clinical data is available for MRT, and the relevance and biological impact of PVDR is not yet fully understood.

Additionally, compared with conventional megavoltage radiotherapy, the LFXT may impose additional limitations (e.g. on beam angle and couch movement) which reduce treatment-planning flexibility. To explore the dosimetric feasibility and potential scope of MRT under ideal conditions, we purposefully did not account for these potential limitations. A new study is currently being prepared that includes these modified machine-specific limitations.

PVDR is considered a key dosimetric parameter in MRT and has traditionally been assumed to influence therapeutic gain by combining enhanced tumour cell kill in the peak regions with minimal damage to normal tissue in the valley regions, thereby improving treatment outcomes [22, 24]. While the valley dose has been shown to correlate most strongly with normal tissue sparing [25], very high peak doses have also been demonstrated to induce acute toxicity [26]. Conversely, peak doses of approximately 150 Gy have been reported to be most effective at inducing tumour growth delay [22], without causing detrimental effects in normal tissue, even at substantially higher peak doses of several hundred Gy in preclinical experiments [3]. Surely, the transferability of discrete dose values from animal experiments to clinical application must be questioned since biological response to radiation depends on species-specific anatomy, physiology, tissue composition, and radiation-response mechanisms. In particular, differences in dose-to-volume across organ sizes and microstructures might affect the transferability of dose values between species.

The maximum valley dose of 7.6 Gy to a limited subvolume of an OAR observed in this study can be considered safe when compared with dose levels routinely tolerated in conventional radiotherapy and is unlikely to cause clinically relevant or severe damage to the majority of OARs. For the selected cases here, a dose level of 7 Gy is likely to result in skin toxicity with erythema and permanent epilation. In contrast, more severe reactions, including moist desquamation, ulceration, and necrosis, generally occur at substantially higher doses [27]. For irradiation of the head near optical structures, the risk of radiation-induced optic nerve and chiasm neuropathy was estimated at 1% for a single dose of 10 Gy [28]. However, other highly radiosensitive structures such as the ocular lenses, cannot be adequately spared when they are in direct proximity to, or overlap with, the target volume. Bony structures are rather robust to radiation exposure, and single-dose delivery is quite common. Doses per fraction ≥ 20 Gy may increase the risk of severe adverse events such as vertebral collapse fracture [29]. For other organs, especially head and neck tumours, data on single-fraction dose delivery are sparse, and no robust clinical single-fraction tolerance threshold has been established for the corresponding OARs.

Notably, a recent comprehensive review by Fernández-Palomo et al. [30] demonstrated that valley dose shows a stronger correlation with survival outcomes in preclinical studies than PVDR, thereby challenging the long-held assumption that PVDR is the primary determinant of MRT effectiveness. Furthermore, in a recent study on proton minibeam irradiation in rats, a higher peak dose of 70 Gy (PVDR = 6.9) was associated with worse neurocognitive outcomes compared with a lower peak dose of 55 Gy (PVDR = 10), highlighting the critical role of peak dose in normal tissue toxicity and indicating that higher PVDR values are not inherently beneficial [31]. The mean peak and valley doses found in this study match the dosimetric values reported in the review above, indicating that our data are consistent with the current literature. However, for clinical translation in a first phase 1 study, utmost importance must be placed on safety. Thus, further reduction of peak doses should be considered.

## Conclusions

This study provides a clinically grounded and dosimetrically validated framework for the first-in-human application of MRT. Using real patient datasets and predefined eligibility criteria, we identified superficially located tumours (particularly in gluteal, nuchal, and inguinal regions) as the most suitable initial indications. We have demonstrated that MRT plans can meet conventional organ-at-risk constraints for these carefully selected scenarios. At the same time, we delineate key physical and technical limitations including restricted beam penetration, reduced planning flexibility, and pronounced spatial dose inhomogeneities which currently limit feasibility in anatomically complex regions. Although the achieved peak, valley, and PVDR values are consistent with preclinical experience, their biological significance and clinical transferability remain incompletely understood, underscoring the need for a conservative, safety-driven dose-escalation strategy. Together, these results define rational entry points for clinical translation and establish a robust foundation for the responsible initiation of phase I MRT trials.

## Data Availability

All relevant data is contained within the article: The original contributions presented in the study are included in the article/supplementary material, further inquiries can be directed to the corresponding author.
